# Complete genome sequence of *Levilactobacillus brevis* NSMJ23, makgeolli isolate with antimicrobial activity

**DOI:** 10.1128/mra.01060-23

**Published:** 2024-01-05

**Authors:** Ju-Hyung Jeon, Jun Sung Kim, Z-Hun Kim, Ji Young Jung

**Affiliations:** 1 Microbial Research Department, Nakdonggang National Institute of Biological Resources (NNIBR), Sangju-si, Gyeongsangbuk-do, South Korea; 2 Hu evergreen Pharm Corp., Bupyeong-gu, Incheon, South Korea; Rochester Institute of Technology, USA

**Keywords:** *Levilactobacillus brevis*, makgeolli isolate, probiotic lactic acid bacteria (LAB), complete genome sequence

## Abstract

We report the complete genome sequence of *Levilactobacillus brevis* NSMJ23 with probiotic properties. The final genome assembly consisted of a 2,389,998-bp chromosome and seven plasmids with 45.59% GC content, which comprised 2,624 genes including 2,457 protein coding sequences.

Lactic acid bacteria (LAB) constitutes economically important bacteria in health and food and feed industries (1, 2). *Levilactobacillus brevis* is a heterofermentative member of the LAB group, which has been isolated from different sources such as human intestine, cheese, Korean kimchi, and brewery, and it is considered to be a potentially probiotic type of LAB due to its host health properties. *L. brevis* strains have been reported to possess various beneficial effects as probiotics, including intestinal microbiota homeostasis (3, 4), modulation of inflammatory and immune responses (5, 6), skin health improvement (7), and anti-obesity (8). The member of *Lactobacillus brevis* appears to be efficient in the production of gamma-aminobutyric acid (GABA) that provides multiple health benefits (9, 10). In our recent research, we have shown that *L. brevis* NSMJ23, a makgeolli (Korean traditional fermented liquor) isolate, can exhibit antimicrobial properties against gut pathogens due to its probiotic property (11). Here, we present the complete genome sequence of *Levilactobacillus brevis* NSMJ23 to analyze its genetic characters.

To obtain the genome sequence, the genomic DNA (gDNA) was extracted from cells of the strain NSMJ23 cultured in MRS (de Man Rogosa and Sharpe) broth (Difco, USA) at 30°C for 48 h using a Maxwell 16 DNA purification kit (Promega, USA). The combination of PacBio RSII platform and Illumina HiSeq X Ten platform (2 × 151 bp) was applied as a strategy to achieve a high-quality genome assembly. Intact gDNA was sheared to approximately 20 kb fragments using g-TUBE (Covaris, Inc., USA) and purified using AMPure PB magnetic beads, and a sequencing library was constructed by using the PacBio SMRTbell template prep kit 1.0 (PacBio). The intact gDNA (100 ng) was sheared using a LE220 focused-ultrasonicator (Covaris, Inc.) and a sequencing library was prepared using the TruSeq Nano DNA library prep kit (Illumina), yielding approximately 350 bp inserts. As for PacBio sequencing data, a total of 152,326 subreads (1.48 Gbp, 565.65-fold coverage; mean subread length, 9,715 bp; N50, 152,326 bp) were generated by removing the adapter sequence and low-quality reads by SMRTlink (v7.0) (12). These high-quality reads were *de novo* assembled using CANU v1.7 (correctedErrorRate = 0.015) (13). A total of 6,213,446 quality-filtered Illumina paired-end reads (0.94 Gbp, 358.61-fold coverage), in which 90% of the bases had a phred score of 30 or higher, were obtained for the error correction step. The PacBio assembly genome was corrected using the Illumina short reads through Pilon v1.21 (14). Default parameters were used except where otherwise noted.

The final genome assembly consisted of a 2,616,331 bp circular chromosome and eight plasmids (pLBN-1, pLBN-2, pLBN-3, pLBN-4, pLBN-5, pLBN-6, and pLBN-7) with a mean sequencing depth of 332× and GC content of 45.59%. The genome sequencing data statistics are summarized in Table 1. NCBI (National Center for Biotechnology Information) Prokaryotic Genome Annotation Pipeline 6.3 (15) identified 2,457 protein-coding genes, 15 rRNA genes, 66 tRNA genes, 3 ncRNAs, and 83 pseudogenes. Several cell-surface proteins (sortase *SrtA*) and LPXTG-motif cell wall-anchoring proteins, which were found to adhere to gastric epithelial cells and could result in the ability to provide a physical barrier to prevent pathogens (16), were detected in the genome. D-alanyl-lipoteichoic acid biosynthesis proteins (dltD), which were involved in the immunobiotic effects on prevention against inflammatory damage (17), were detected. A glutamate decarboxylase that catalyzes the production of GABA was detected in the genome. Its amino acid sequence had high percent identity (90.9%–100%) with those of known *L. brevis* strains upon NCBI-BLASTP (protein-protein BLAST). The antiSMASH (v7.0.1) (18) and BLASTP predicted a lanthipeptide synthetase LanC family protein (19). This whole genome sequence will provide a better understanding of the potential functionality of the potential probiotic *Levilactobacillus brevis* NSMJ23.

**TABLE 1:** Summary of assembly and annotation statistics of the *Levilactobacillus brevis* strain NSMJ23

Genetic element	Genome size (bp)	GC contents (%)	No. of coding sequences	No. of rRNAs	No. of tRNAs	Sequencing depth (×)	GenBank accession no.
Chromosome	2,389,998	46.12	2,207	15	64	287	CP050541
pLBN-1	53,263	38.76	53	0	0	1,508	CP050542
pLBN-2	44,181	41.64	57	0	2	625	CP050543
pLBN-3	41,855	40.92	44	0	0	536	CP050544
pLBN-4	36,959	39.62	39	0	0	791	CP050545
pLBN-5	21,191	39.30	24	0	0	730	CP050546
pLBN-6	14,686	40.24	15	0	0	359	CP050547
pLBN-7	14,198	38.98	18	0	0	207	CP050548
Total	2,616,331	45.59	2,457	15	66	332	

## Data Availability

The genome sequence and raw sequencing reads for strain NSMJ23 were deposited under GenBank accession numbers CP050541, CP050542, CP050543, CP050544, CP050545, CP050546, CP050547, and CP050548, BioProject accession number PRJNA614607, BioSample accession number SAMN14433864, and SRA accession numbers SRX22644591 and SRX22644592
